# Overexpression of c-Met is Associated with Poor Prognosis in Glioblastoma Multiforme: A Systematic Review and Meta-Analyses

**DOI:** 10.31557/APJCP.2021.22.10.3075

**Published:** 2021-10

**Authors:** Jellyca Anton, Sudibio Sudibio, Handoko Handoko, Tiara Bunga Mayang Permata, Henry Kodrat, Endang Nuryadi, Henry Sofyan, Eka Susanto, Rahmad Mulyadi, Renindra Ananda Aman, Soehartati Gondhowiardjo

**Affiliations:** 1 *Department of Radiation Oncology, Dr. Cipto Mangunkusumo Hospital, Jakarta, Indonesia. *; 2 *Faculty of Medicine, Universitas Indonesia, Jakarta, Indonesia. *; 3 *Department of Neurology, Dr. Cipto Mangunkusumo Hospital, Jakarta, Indonesia.*; 4 *Department of Pathology, Dr. Cipto Mangunkusumo Hospital, Jakarta, Indonesia. *; 5 *Department of Radiology, dr. Cipto Mangunkusumo Hospital, Jakarta, Indonesia. *

**Keywords:** c-Met, glioblastoma multiforme, prognosis, survival

## Abstract

**Objective:**

The aim of this study is to evaluate the association of c-Met overexpression with survival of glioblastoma multiforme (GBM) patients.

**Methods:**

A systematic review with meta-analyses was conducted on related articles from PubMed, EBSCOhost, Scopus, and Cochrane databases with last updated search on October 31, 2020. A total of 7 studies regarding c-Met overexpression and overall survival (OS) and/or progression free survival (PFS) are included in this study.

**Results:**

All studies used immunohistochemistry to examine the expression of c-Met protein. The results showed that the positive rate of c-Met overexpression was detected in approximately 33,9% - 60,5% of GBM patients. c-Met overexpression was related to worse OS (HR: 1,74; 95% CI: 1,482-2,043; Z=6,756; p<0,001) and PFS (HR: 1,66; 95% CI: 1,327-2,066; Z=4,464; p<0,001) in GBM patients. Low heterogeneity of subjects was found in both OS and PFS analyses, I^2^ values were 7,8% and 0,0%, respectively.

**Conclusion:**

In conclusion, c-Met overexpression is significantly related to shorter OS and PFS in GBM patients, so c-Met can be considered as a potential prognostic indicator in GBM.

## Introduction

Glioblastoma multiforme (GBM) is a highly aggressive brain malignancy originating from glial cells and is classified as grade IV glioma according to WHO classification of CNS tumours 2016 (Louis et al., 2016). According to Central Brain Tumour Registry of the United States (CBTRUS), GBM accounts for 14,5% of all primary brain tumour cases and 80% of all high grade gliomas. The incidence rate was 3,23 per 100,000 populations and more common in male compared to female (Ostrom et al., 2018). In spite of the current standard treatment protocol, including maximal safe resection followed by concurrent chemoradiotherapy and adjuvant chemotherapy with the alkylating agent temozolamide (TMZ), the prognosis of GBM patients remains poor. The median survival for the newly diagnosed GBM patients with this therapy paradigm is approximately 12,1-14,6 months (Stupp et al., 2009).

Clinical prognostic factors that contribute to the survival in this devastating disease were firstly studied by Radiation Therapy Oncology Group (RTOG) in 1993, using Recursive Partitioning Analysis (RPA) based on patient, tumour, and therapy variables (Curran et al., 1993). In 2011, this RPA model were validated and simplified and three prognostic classes (class III, IV, V-VI) were establised according to age, Karnofsky Performance Status (KPS), and extent of resection (Li et al., 2011). This new RPA model is then frequently used in many clinical settings. However, similar to other malignancies that harbor molecular disregulation processes, there are also several molecular pathways responsible for the pathogenesis of GBM (Kresno and Gondhowiardjo, 2004). Until now, many studies had been conducted in order to find which molecular pathways that could predict the prognosis of GBM patients (Bell et al., 2017).

One of the most interesting moleculars is the receptor tyrosine kinase c-Met (mesenchymal-epithelial transition), which is coded by proto-oncogene MET located in chromosome 7q21-31 (Liu et al., 2011). c-Met is expressed in epithelial and endothelial cells, liver, pancreas, prostate, kidney, bone marrow, and at low levels in the brain (Cruickshanks et al., 2017; Petterson et al., 2015). Its primary ligand is called hepatocyte growth factor (HGF). c-Met/HGF signalling pathway plays an important role in normal biological functions such as embryogenesis, tissue regeneration, wound healing, and formation of nerve and muscle (Zhang et al., 2018). This pathway has also been known to involve in tumorigenesis by inducing cell survival, proliferation, adhesion, migration, invasion, anti-apoptotic responses, and angiogenesis (Liu et al., 2011; Olmez et al., 2013). Abnormal activation of c-Met (by gene amplification, mutation, transloction, or auto-/paracrine ligand signaling) has been linked to the development and progression of many types of cancer, including hepatocellular carcinoma, lung cancer, colorectal cancer, breast cancer, pancreatic cancer, ovarian cancer, prostate cancer, gastric cancer, and GBM (Olmez et al., 2013; Zhang et al., 2018).

Growing evidence shows the involvement of c-Met in the GBM pathogenesis. It was suggested that the expression of HGF together with its receptor c-Met stimulated the growth of HGL4 GBM cell lines (Shiota et al., 1996). A research using cell lines indicated the presence of multiple mechanisms of c-Met activation in GBM (Uchinokura et al., 2006). Another study reported that c-Met was important for endothelial mesenchymal transition, aberrant vascularization, cancer progression, and chemoresistance in GBM (Huang et al., 2016). From a clinical standpoint, it had been showed that overexpression of c-Met was detected in 33,9% of patients with GBM, and this significantly associated with shorter overall survival (OS) and progression free survival (PFS) (Kong et al., 2008). According to a study by NRG Oncology RTOG 0525, protein biomarkers significantly correlated with shorter OS after multimarker modeling were c-Met, Ki-67, and MGMT (Bell et al., 2017). On the other hand, it was also reported that the survival of GBM patients was longer in those who harbored c-Met overexpression (Kwak et al., 2015). Because of these contradictive results, we performed a systematic review of literatures with meta analysis to evaluate the prognostic value of c-Met in predicting the survival of patients with GBM.

## Materials and Methods

*Literature search and selection criteria*

This systematic review with a meta-analysis was conducted based on the Preferred Reporting Items for Systematic Reviews and Meta-Analyses (PRISMA) recommendations (Moher et al., 2009). Articles regarding c-Met expression and survival in GBM were systematically searched on PubMed, EBSCOhost, Scopus, and Cochrane databases, using these following keywords: (glioblastoma OR glioblastoma multifor*) AND (CMET OR MET) AND (prognos* OR surviv*). The search was done from May 1, 2020 until the last updated search on October 31, 2020. Only publications in English were collected with no restriction on the year of publication. Studies were included in this review if these following criteria were met: (1) articles were available in full paper; (2) expression of c-Met protein expression was assessed and was associated with the survival (OS and PFS) of GBM patients; (3) cut-off value for c-Met protein overexpression was stated; (4) data on hazard ratio (HR) and 95% confidence interval (95% CI) were provided or could be calculated from the sufficient data.

*Data extraction*

Review of all articles and extraction of the data needed were done by two reviewers independently. Disagreements were discussed and resolved among the authors. Information recorded from each study including name of the first author, year of publication, region, sample size, c-Met protein cut-off value, c-Met overexpression positive rate, OS and PFS data, and HR with 95% CI.

*Statistical analysis*

In every study, the OS and/or PFS of GBM patients were compared between the groups with and without c-Met overexpression. The result was considered significant if the calculated p-value was <0,05. The intensity of relationship between c-Met expression level and OS or PFS was presented in HR. The overexpression of c-Met predicted shorter survival of GBM patients if the value of HR > 1 and 95% CI not overlapping 1. In some articles, HR with 95% CI was available from univariate or multivariate analysis, and this value could be directly obtained. Otherwise, HR and its corresponding 95% CI were estimated from Kaplan-Meier survival curve using a specific reported method (Tierney et al., 2007). To evaluate the prognostic value of c-Met, the pooled HR corresponding to the 95% CI was calculated using STATA 12.0. Statistical heterogeneity was determined by Cochrane’s Q test (Chi-squared test; Chi2) and inconsistency (I^2^). The pooled HR was estimated by the fixed-effects model if no obvious heterogeneity was found. Otherwise, the random-effects model was used.

## Results

*Studies selection and characteristics*

**Figure 1:**
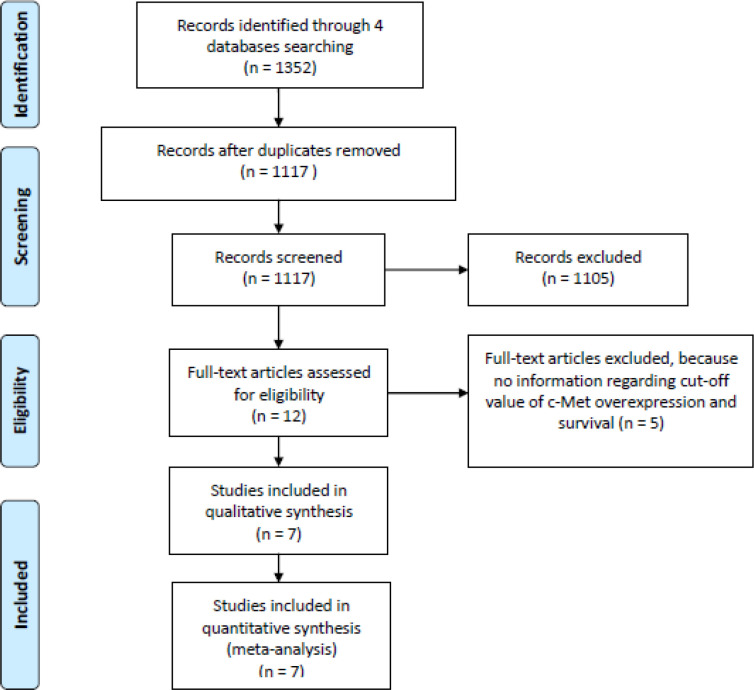
The Flow Diagram of Study Selection Process

Using the specified keywords mentioned beforehand, a total of 1352 studies was obtained from 4 databases. The flow diagram of the study selection process can be seen in Figure1. Titles and abstracts of the published articles were screened by 2 reviewers independently. Following deduplication, 12 studies were agreed to be retrieved for detailed review and the full texts were collected. Five out of 12 studies were excluded after careful review of the study methodologies because no information regarding cut-off value of c-Met overexpression and survival. Finally, 7 studies were included in this review (Kong et al., 2008; Liu et al., 2010; Olmez et al., 2013; Petterson et al., 2015; Li et al., 2016; Bell et al., 2017; Ohba et al., 2019).

Studies were carried out in 7 different countries (5 in Asia, 1 in America, 1 in Europe) from 2008-2019 using retrospective cohort. The main information of the articles can be seen in Table1 and Table2. The sample sizes ranged from 19 to 452 patients. Immunohistochemistry was used to assess c-Met expression in all studies, with positive rate approximately 33,9% - 60,5%. The cut-off values varied, with >30% being the most frequently used. Six studies examined the association of c-Met expression with OS in GBM, all of them demonstrated that overexpression of c-Met significantly related to shorter OS. In these studies, 4 articles provided the multivariate HRs, 1 reported univariate HR, and 1 showed Kaplan-Meier survival curves that could be used to calculate HRs. The results of all 5 studies evaluating PFS demonstrated that PFS was significantly shorter in GBM patients who harbored c-Met overexpression. Hazard ratio from multivariate analysis was available in one study, whereas HRs from 4 other studies were calculated from the Kaplan-Meier survival curves. 

*Meta-analysis*

**Figure 2:**
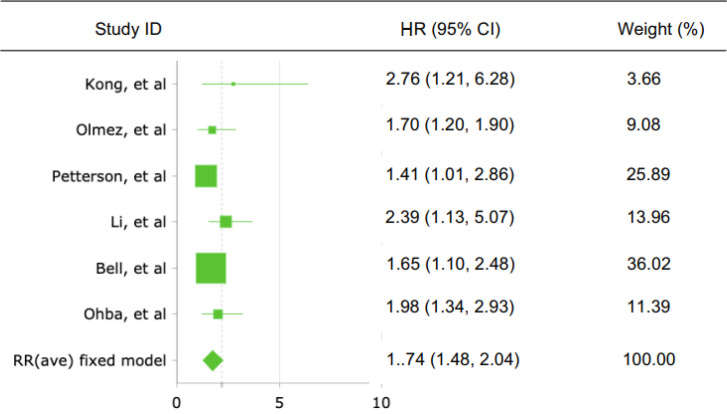
Forest Plot Showing the Combined Hazard Ratio (HR) from Fixed-effect for Overall Survival

**Figure 3:**
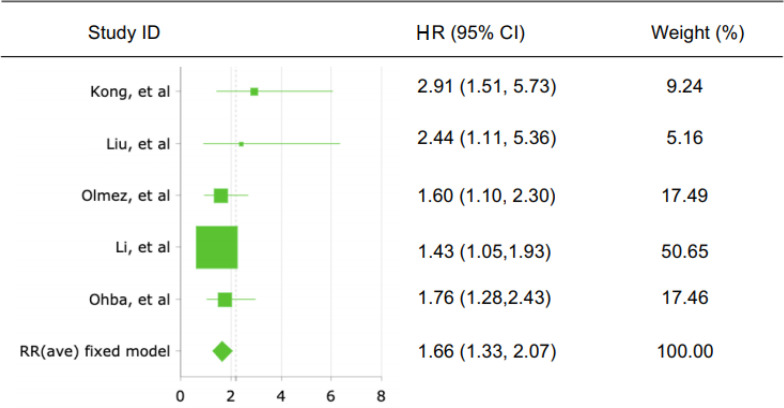
Forest Plot Showing the Combined Hazard Ratio (HR) from Fixed-effect for Progression Free Surviva

From the meta-analysis of 6 studies assessing c-Met expression and OS, the pooled HR was 1,74 (95% CI: 1,482-2,043; Z=6,756; p<0,001, Figure 2) with heterogeneity I2 = 7,8%; p<0,001. In studies evaluating PFS, the pooled estimate of risk was 1,66 (95% CI: 1,327-2,066; Z=4,464; p<0,001, Figure 3) with heterogeneity I^2^ = 0,0%; p<0,001. These results suggested that overexpression of c-Met was significantly associated with shorter OS and PFS of GBM patients, so that high c-Met expression was a valuable molecular prognostic factor for OS and PFS in GBM. Low heterogeneity of subjects was found in both OS and PFS analyses (both I^2^ values < 50%).

**Table 1:** Information from Studies on c-Met Expression and OS in GBM Patients

Author	Year	Region	Number of patients	IHC cut-off	Positive rate	OS (with vs without c-Met overexpression)	HR	95% CI	p
Kong et al.	2008	South Korea	62	>25%	33.90%	Median OS 10,7 vs 20 months; p<0,001	2,761 (multivariate)	1,214-6,280	0.015
Olmez et al.	2013	Turkey	69	>30%	45%	Mean OS 15,3 ± 2,3 vs 22,6 ± 2,5 months; p<0,01	1,7 (multivariate)	1,2-1,9	<0,01
Petterson et al.	2015	Denmark	186	>75%	40%	Overexpression of c-Met is significantly associated with shorter OS	1,41 (univariate)	1,01-1,86	0.03
Li et al.	2016	China	175	>30%	60.50%	Mean OS is shorter in patients with c-Met overexpression	2,389 (multivariate)	1,126-5,072	0.023
Bell et al.	2017	USA	452	Top quartile	ND	Overexpression of c-Met is significantly correlated with decreased OS	1,65 (multivariate)	1,10-2,48	0.02
Ohba et al.	2019	Japan	59	>30%	52.50%	Median OS 14,7 vs 23,4 months; p=0,018	1,98 (survival curve)	1,43-2,93	<0,05

IHC, immunohistochemistry; OS, overall survival; c-Met, mesenchymal epithelial transition; HR, hazard ratio; CI, confidence interval; p, p-value; ND, no data

**Table 2:** Information from Studies on c-Met Expression and PFS in GBM Patients

Author	Year	Region	Number of patients	IHC cut-off	Positive rate	PFS (with vs without c-Met overexpression)	HR	95% CI	p
Kong et al.	2008	South Korea	62	>25%	33.90%	Median PFS 3,2 vs 9,2 months; p=0,002	2,91 (survival curve)	1,51-5,73	0.014
Liu et al.	2010	China	19	>30%	36.80%	Median PFS 6,1 vs 11,5 months; p=0,026	2,44 (survival curve)	1,11-5,36	<0,05
Olmez et al.	2013	Turkey	69	>30%	45%	Mean PFS 12,3 ± 2,1 vs 19,1 ± 2,6 months; p<0,05	1,6 (multivariate)	1,1-2,3	<0,05
Li et al.	2016	China	175	>30%	60.50%	Mean PFS was shorter in patients with c-Met overexpression	1,43 (survival curve)	1,05-1,93	0.01
Ohba et al.	2019	Japan	59	>30%	52.50%	Median PFS 5,3 vs 8,3 months; p=0,045	1,76 (survival curve)	1,28-2,43	<0,05

IHC, immunohistochemistry; OS, overall survival; c-Met, mesenchymal epithelial transition; HR, hazard ratio; CI, confidence interval; p, p-value

## Discussion

GBM is a devastating disease with high mortality and high recurrence rates. While clinical prognostic factors including age, KPS, and extent of resection had been well established, many studies had been conducted to find the possible molecular prognostic factors involved in GBM (Curran et al., 1993; Li et al., 2011). The receptor tyrosine kinase c-Met that binds to its ligand HGF is one of the exprected moleculars. c-Met/HGF has been known to connected with many physiological and pathological process (Zhang et al., 2018). Deregulation of c-Met expression has been linked to the development, progression, and prognosis of many types of human cancers, such as hepatocellular carcinoma, colorectal cancer, ovarian cancer, breast cancer, gastric cancer, and osteosarcoma (Kong et al., 2008; Ohba et al., 2019; Zhang et al., 2018). c-Met/HGF signaling could induce proliferation, motility, cell adhesion, invasion, and anti-apoptosis. This pathway could also increase tumourigenicity and malignancy progression by activating cell cycle progression, cell migration, and tumour angiogenesis (Kong et al., 2008). 

The result of this systematic review with a meta analysis demonstrated that overexpression of c-Met was associated with worse OS and PFS in GBM patients. Interaction of c-Met and HGF on the cell surface leads to the autophosphorylation of Tyr1234 and Tyr1235 in the catalytic site, followed by phosphorylation of the tyrosine residue Tyr 1349 and Tyr 1356 in the docking site of the receptor (Zhang et al., 2018). This processes trigger the recruitment and activation of many signaling effectors, such as Gab1 (Grb2-associated adaptor protein 1), Grb2 (growth factor receptor-bound protein 2), Src, Shc (Src homology domain c-terminal adaptor homolog), Shp2 (Src homology protein tyrosine phosphatase 2), PLC-γ (phospholipase c-γ), FAK (focal adhesion kinase); followed by the phosphorylations of transducers, including STAT3 (signal transducer and activator of transcription 3), Ras/MAPK/ERK, PI3K (phosphatidylinositol 3-kinase)/Akt (Cruickshanks et al., 2017). These signals have been implicated in survival, proliferation, invasion, migration, angiogenesis, and stemness of glioma cells. Under normal conditions, cytoplasmic Tyr 1003 works as the negative regulator of c-Met that will recruit c-CBL, and phosphorylation of c-CBL leads to receptor degradation (Cheng and Guo, 2019). 

A research using cell lines showed the existence of multiple mechanisms of c-Met activation in GBM (Uchinokura et al., 2006). It had been demonstrated that c-Met was responsible for endothelial mesenchymal transition, aberrant vascularization, cancer progression, and chemoresistance in GBM (Huang et al., 2016). The overexpression of c-Met was also significantly associated with higher molecular phenotypes that represent invasiveness, MMP-2 and MMP-9, and tended to show invasive and multifocal features on the initial magnetic resonance images (Kong et al., 2008). From a result from an in-vitro research, a decrease in c-Met expression caused inhibition of cell migration and invasion capacity, and also increased the sensitivity to TMZ (Li et al., 2016). In addition, it was reported that c-Met overexpression was more frequently found in recurrent GBM compared to primary GBM (Liu et al., 2011). 

All articles in this review used immunohistochemistry to detect the overexpression of c-Met. It could be seen that the positive rate of c-Met overexpression was quite high in each study (33,9% - 60,5%). The cut-off values to define c-Met overexpression varied. Most studies used > 30% as cut-off value (Li et al., 2016; Liu et al., 2011; Ohba et al., 2019; Olmez et al., 2013). One study took 75% as cut-off after it was found that cut-off 70,8% (from median) was not prognostic (Petterson et al., 2015). According to a study with large sample size by NRG Oncology RTOG 0525, the level of c-Met protein expression within the cytoplasm when split by the top-tertile and top-quartile was significantly associated with shorter OS, with value from top-quartile reached significancy after a multimarker model. However, the specific number of cut-off value was not mentioned in this study (Bell et al., 2017).

This article is the first meta-analysis that evaluates c-Met overexpression and survival in patients with GBM. In conclusion, c-Met overexpression is significantly related to shorter OS and PFS in GBM patients, so c-Met can be considered as a potential prognostic indicator in GBM. This study could also propose that c-Met targeted therapy in GBM may be promising.

## Author Contribution Statement

Authors’ contributions including conceptualization, S.G, R.A.A, R.M, E.S, H.S; methodology, S.G, J.A, S, H, T.B.M, H.K, E.N; validation, J.A, S; formal analysis, J.A, S, S.G; data curation, J.A, S, H, T.B.M, H.K, E.N; writing-original draft preparation, J.A, S, H; writing-review and editing, J.A, S, S.G, H, T.B.M, H.K, E.N. All the authors declare no conflict of interest.
